# Risk factors for and protective factors against breastfeeding interruption before 2 years: a birth cohort study

**DOI:** 10.1186/s12887-021-02777-y

**Published:** 2021-07-09

**Authors:** Graciete Oliveira Vieira, Tatiana de Oliveira Vieira, Camilla da Cruz Martins, Michelle de Santana Xavier Ramos, Elsa Regina Justo Giugliani

**Affiliations:** 1 Universidade Estadual de Feira de Santana, Feira de Santana, BA Brazil; 2 Universidade Federal do Recôncavo da Bahia, Santo Antônio de Jesus, BA Brazil; 3 Universidade Federal do Rio Grande do Sul, Porto Alegre, RS Brazil

**Keywords:** Breastfeeding, Social determinants of health, Child nutrition, Child health

## Abstract

**Background:**

Little is known about the factors associated with the World Health Organization (WHO) recommendation of breastfeeding for at least 2 years. The objective of this study was to identify risk factors for and protective factors against breastfeeding interruption before 2 years of age.

**Methods:**

In this live birth cohort, mother and infant dyads were followed for 2 years. Data collection was performed at the maternity ward and subsequently at the children’s homes, monthly during the first 6 months of life and then at 9, 12, 18, and 24 months. The outcome of interest was breastfeeding interruption before 2 years of age. Median duration of breastfeeding was estimated using Kaplan-Meier’s survival analysis, and the associations were tested using Cox’s hierarchical multivariate model. Significance was set at 5%.

**Results:**

Data from a total of 1344 dyads were assessed. Median breastfeeding duration was 385 days. The following risk factors for breastfeeding interruption were identified: white skin color (adjusted hazard ratio [HRa]: 1.31; 95% confidence interval [95%CI]: 1.10–1.56), primiparity (HRa: 1.21; 95%CI: 1.05–1.40), working outside the home (HRa: 1.52; 95%CI: 1.30–1.77), child sex male (HRa: 1.18; 95%CI: 1.03–1.35) and use of a pacifier (HRa: 3.46; 95%CI: 2.98–4.01). Conversely, the following protective factors were identified: lower family income (HRa: 0.81; 95%CI: 0.71–0.94), mother-infant bed-sharing (HRa:0.61, 95%CI: 0.52–0.73), on-demand breastfeeding in the first month (HRa: 0.64; 95%CI: 0.47–0.89) and exclusive breastfeeding at 4 months (HRa: 0.58, 95%CI: 0.48–0.70).

**Conclusions:**

The findings allowed to identify both risk factors for and protective factors against breastfeeding interruption before 2 years of age. Knowledge of these factors may help prevent this event and aid in the development of programs that help women maintain breastfeeding for at least 2 years, as recommended by the WHO.

## Background

The benefits of breastfeeding have been supported and confirmed by robust scientific evidence, with impact on reducing infant mortality due to infectious diseases [1]. Moreover, some of those benefits have been shown to have a dose-response effect [2–4], such as protection against asthma [2, 5], overweight/obesity in adolescence and adulthood, diabetes type 2 [6], dental malocclusion [4], in addition to a positive impact on cognitive development [7, 8]. Breastfeeding also has advantages to the health of the lactating woman, e.g. protecting against breast and ovarian cancer, and diabetes type 2, and increasing the period of lactational amenorrhea [5].

The World Health Organization (WHO) recommends breastfeeding for 2 years or more; breastfeeding should be exclusive in the first 6 months, and subsequently supplemented with other healthy foods [9]. However, in Brazil, the prevalence of breastfeeding maintenance is low, with rates of 47.5% at 12 months and 24.8% at 2 years [10]. Worldwide, breastfeeding maintenance is more frequent in low- and middle-income countries, reaching rates around 90% for breastfeeding maintenance at 12 months and 60% at 2 years in lower-income countries [11]. In most high-income countries, less than 20% of the children are breastfed until 1 year of age [11].

Little is known about the risk factors for and protective factors against breastfeeding interruption before 2 years of age. To date, five articles have been published addressing this topic: two Brazilian studies [12, 13], one Croatian study [14], one from the United States [15] and one carried out in Indonesia [16]. In the Croatian study [14], which recruited women from only one maternity hospital, those with low education level, who had not attended a prenatal course and who had not been instructed on how often to feed their children had a higher risk of abandoning breastfeeding before of 2 years. In the Indonesian study [16], for housewives in a rural area the risk factors were: mothers’ plan to breastfeed for less than 2 years, mothers’ belief that breastfeeding less than 2 years was the norm and exposure to exclusive breastfeeding promotion. In Los Angeles [15], among women’s WIC participants, those who did not have the intention to breastfeed prior to childbirth, did not breastfeed in the maternity, were not interviewed in Spanish, and did not return to work in the first 3 months were at greater risk for the outcome. And in Brazil, both studies [12, 13] were conducted with women delivering in a maternity in the south of the country, but one [13] included only adolescent mothers. In the study that included mothers of all ages [12], the following factors were negatively associated with maintenance of breastfeeding for 2 years or more: cohabiting with the infant’s father, mother working outside home in the first semester after childbirth, child using a pacifier and earlier introduction of water, tea, and complementary feeding to the infant’s diet. The only factor common to both studies was using a pacifier. In addition to the use of a pacifier, primiparity and younger father’s age put the adolescent mothers at greater risk of weaning before 2 years of age [13]. Thus, the results of these five studies showed that factors associated with breastfeeding maintenance for 2 years or more differed greatly across the studies, pointing to the need for continued investigation to improve our understanding of this phenomenon.

Taking into account the diversity of risk factors associated with weaning before the child’s 2 years of age in the existing studies and the fact that none of these studies was conducted with a representative population, the objective of this study was to identify risk factors for and protective factors against breastfeeding interruption before 2 years of age in a birth cohort involving all maternities of a medium-sized city in northeast Brazil, a region with different social, economic and cultural characteristics compared to the south of the country, where the previous Brazilian studies were conducted.

## Methods

This was a cohort of live births in which the mother-infant dyads were followed until 2 years of child age. The study was carried out in Feira de Santana, a municipality in the countryside of the state of Bahia, with a population estimated at 609,913 inhabitants at year 2018 [17].

### Participants and sample size calculation

Sample selection was conducted between April 2014 and March 2015 at the 10 maternities located in the municipality of Feira de Santana, state of Bahia. For 2 consecutive months, women were recruited from each maternity. Data collection was performed simultaneously at two maternities each time, except for two hospitals that had a high number of births and were therefore handled individually. The order of hospital selection was determined by drawing lots.

Inclusion criteria were: women who initiated breastfeeding in the maternity, who did not present complications during pregnancy or after childbirth, and who gave birth to newborns without perinatal complications. The following criteria determined exclusion from participation in the study: newborns with congenital malformations or admitted to the neonatal unit for more than 24 h, mother or child with contraindications for breastfeeding, and mothers separated from their children due to judicial enforcement.

Sample size calculation resulted in 1183 mother-infant dyads, considering the number of live births registered in the municipality in 2003 (10,177), the proportion of children breastfed for 2 years or more (32.5%) [12], a level of significance of 5%, a test power of 90%, and a difference of at least 5% in the outcome, corrected for a finite population.

### Data collection

Data on socioeconomic and demographic characteristics, prenatal care, childbirth, postpartum and newborn periods of the mother-infant dyads were collected via interviews with the mothers at the maternity ward; and data on infant feeding patterns, mother working outside the home, infant using a pacifier, and sleeping environment were obtained monthly at the mothers’ homes in the first 6 months of life, and then at 9, 12, 18, and 24 months of child age. Interviews were conducted by previously trained health professionals, using a structured questionnaire especially designed for the study. At each home visit, the mother-baby dyads not found were considered lost to follow-up.

### Data analysis

All data collected were entered twice into a database using the Statistical Package for the Social Sciences (SPSS) for Windows version 16.0 (Chicago, II, USA) and subjected to validation for the correction of any inconsistencies identified. Statistical analyses were performed using SPSS version 16.0 and the R 15.1 statistical software. Median duration of breastfeeding was estimated using survival analysis with the Kaplan-Meier method.

The outcome of interest was breastfeeding interruption, i. e., when the woman stopped breastfeeding permanently before the child turns 2, considering the mother’s answer to the question: “Has your child breastfed in the last 24 h? [yes, no]. The following explanatory variables were taken into consideration: maternal socioeconomic and demographic characteristics (self-reported skin color [white, non-white], age upon childbirth [< 20, ≥ 20 years], schooling [≤ 8, > 8 years of formal education], parity [primiparous, multiparous], family income [< 2, ≥ 2 minimum wages]); characteristics of the prenatal period (attendance to lecture or course on breastfeeding [yes, no], smoking during pregnancy [yes, no], type of prenatal care funding [public or private/medical insurance plan]); characteristics of the delivery and immediate postpartum period (type of delivery [vaginal, cesarean section], type of hospital funding [public or private/medical insurance plan], breastfeeding in the first hour of life [yes, no], rooming-in at hospital [yes, no], receiving guidance on breastfeeding in the hospital [yes, no], maternal expectation of breastfeeding duration [≥ 12, < 12 months]); newborn characteristics (birth weight [< 2500, ≥ 2500] and sex [male, female]); and variables related to the period following hospital discharge, focusing on both the mother (working outside the home at the time of breastfeeding interruption [yes, no], partner’s support and encouragement to breastfeeding [yes, no], cohabitation with child’s father [yes, no]) and the child (use of a pacifier at the time of breastfeeding interruption or at 24 months for those being breastfed at this time [yes, no]), sleeping environment mother-infant [bed-sharing or not] at the time of breastfeeding interruption or at 24 months for those being breastfed at this time, breastfeeding scheme in the first month of life [on demand, scheduled], and breastfeeding pattern at 4 months of life [exclusive breastfeeding or not]). The rationale of choosing this timepoint for exclusive breastfeeding was the very low prevalence of exclusive breastfeeding beyond this age. Because some of the data collected varied throughout time, specific questions were repeated at all home visits, e.g., infant feeding patterns, mother working outside the home, infant using a pacifier, and sleeping environment.

**Fig. 1:**
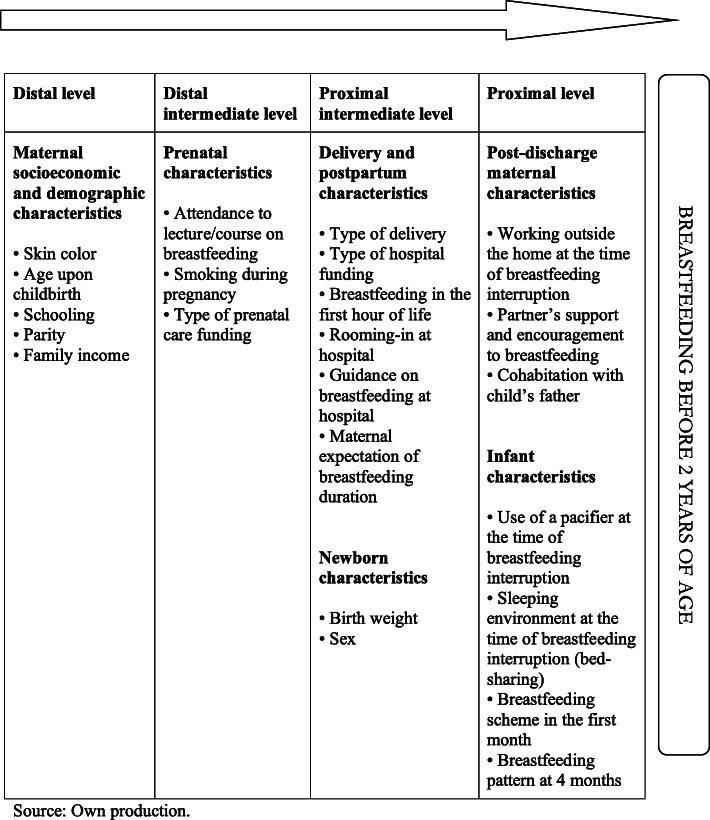
Hierarchical levels used in the multivariate analysis. Source: Own production

Explanatory variables were grouped into four levels according to the hierarchical theoretical model adopted in a systematic review designed to summarize information on factors associated with breastfeeding duration for 12 months or longer, according to their proximity to the outcome [18]. Maternal socioeconomic and demographic variables were included in the distal level, followed by prenatal variables (distal intermediate level), variables related to delivery and immediate postpartum period (proximal intermediate level), and finally variables occurring after hospital discharge (proximal level) (Fig. 1).

Associations between independent variables and the outcome were tested using Cox’s multivariate extended model (fixed or time-variable); factors belonging to each model were entered according to the hierarchical levels established. All variables related to each level were adjusted among themselves, and those showing significant association with the outcome at 5% were selected to remain in the next levels. As a result, variables in the distal intermediate group were adjusted among themselves and also for distal group variables showing significant association with the outcome, and so on successively until the inclusion of the last group of variables (proximal). The theoretical hierarchical model adopted made it possible to evaluate how variables in the same group compete with each other and how more proximal variables may mediate the effects of variables in a preceding group. At the end of the process, risk factors for and protective factors against breastfeeding interruption before 2 years of age were determined. The level of association was estimated using crude hazard ratio (HR) and adjusted hazard ratio (HRa) and their respective 95% confidence intervals (95%CI). Significance was set at *p* < 0.05.

The characteristics of the participants considered as loss were compared with those who completed the follow-up by the sensitivity analysis. In this stage, the level of significance adopted was 5%.

This study safeguarded the ethical principles regarding human research provided for in the Declaration of Helsinki and Resolution 466/12 of the National Health Council of the Ministry of Health/Brazil. It was approved by the research ethics committee of Universidade Estadual de Feira de Santana (protocol no. 077/2006; CAAE 0074.0.059.000–06). Mothers were interviewed after agreeing to participate in the study and signing an informed consent form.

## Results

A total of 1344 mother-infant dyads were included in the study. Two hundred ninety-eight were lost to follow-up (22.1%); of the latter, 233 (17.3%) were lost before the sixth month, 18 (1,3%) between 6 and 9 months, 21 (1,6%) between 9 and 12 months and 26 (1.9%) between 12 and 24 months.

Sample characteristics are described in Table 1. The high frequency of breastfeeding on demand in the first month stands out (95.8%), as does the low prevalence of breastfeeding in the first hour of life (48.0%) and of exclusive breastfeeding at 4 months (21.0%).

**Table 1:** Characteristics of the 1344 mother-infant dyads included in the study, Feira de Santana, Bahia, Brazil

Characteristics	***N***	%
Maternal skin color		
White	245	18.2
Non-white	1099	81.8
Maternal age upon childbirth (years)		
< 20	262	19.5
≥ 20	1082	80.5
Maternal schooling (years of formal education)		
≤ 8	508	37.8
> 8	836	62.2
Parity		
Primiparous	671	49.9
Multiparous	673	50.1
Family income (minimum wages)^a^		
< 2	735	54.7
≥ 2	609	45.3
Attendance to prenatal lecture/course on breastfeeding		
Yes	347	25.8
No	997	74.2
Smoking during pregnancy		
Yes	32	2.4
No	1312	97.6
Type of prenatal care funding		
Public	888	66.1
Private/medical insurance plan	456	33.9
Type of delivery		
Vaginal	759	56.5
Cesarean section	585	43.5
Type of hospital funding		
Public	1019	75.8
Private/medical insurance plan	325	24.2
Breastfeeding in the first hour of life		
Yes	645	48.0
No	699	52.0
Rooming in at hospital		
Yes	1229	91.4
No	115	8.6
Guidance on breastfeeding at hospital		
Yes	1094	81.4
No	250	18.6
Maternal expectation of breastfeeding duration (months)		
≥ 12	230	17.1
< 12	1114	82.9
Birth weight (g)		
< 2500	65	4.8
≥ 2500	1279	95.2
Infant sex		
Male	719	53.3
Female	625	46.5
Mother working outside the home at the time of breastfeeding interruption		
Yes	356	26.5
No	988	73.5
Partner’s support and encouragement to breastfeeding		
Yes	1274	94.8
No	70	5.2
Cohabitation with child’s father		
Yes	1145	85.2
No	199	14.8
Use of a pacifier at the time of breastfeeding interruption		
Yes	564	42.0
No	780	58.0
Sleeping environment at the time of breastfeeding interruption		
Mother-infant bed-sharing	1045	77.8
Other	299	22.2
Breastfeeding scheme in the first month^b^		
On demand	1236	95.8
Scheduled	54	4.2
Breastfeeding pattern at 4 months^c^		
Exclusive breastfeeding	255	21.0
Non-exclusive breastfeeding	962	79.0

^a^ 1 minimum wage = US$ 117,49

^b^ Total of 1290 dyads analyzed

^c^ Total of 1217 dyads analyzed

It was observed that the participants who composed the losses had characteristics common to those who completed the study, at the level of 5%, except for the variables: family income, parity, mother working outside the home when breastfeeding was interrupted, use of a pacifier at the time of breastfeeding interruption and pattern of breastfeeding at 4 months.

**Fig. 2:**
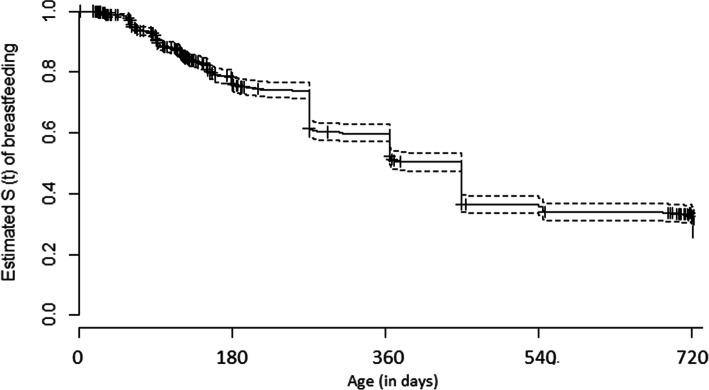
Kaplan-Meier survival curve showing breastfeeding rates up to 2 years of age. Source: Own production

Median breastfeeding duration was 385 days (95%CI: 365–450 days). Figure 2 shows the survival curve for the probability of breastfeeding maintenance until at least 2 years of child age. Breastfeeding survival behavior at 30, 120, 180, and 365 days was, respectively, 99.3, 85.8, 76.1, and 52.1%. The estimated prevalence of breastfeeding at 24 months of age was 20.8%.

In the multivariate analysis, conducted according to the hierarchical model proposed, the following risk factors were identified for breastfeeding interruption before 2 years of age: white skin color, working outside the home, infant sex male, and use of a pacifier. Conversely, family income below two minimum wages, mother-infant bed-sharing, on-demand breastfeeding in the first month after birth and exclusive breastfeeding at 4 months of life were protective factors for breastfeeding maintenance until 2 years or more. No effect modifiers were identified in the analysis. The model showed good adjustment. Table 2 presents the crude and adjusted effects (HR) obtained for each predictor of breastfeeding interruption before 2 years of age.

**Table 2:** Cox’s univariate analysis and Cox’s multivariate regression analysis for risk of breastfeeding interruption before 2 years of age

Variables	Crude HR (95%CI)	Distal level HRa (95%CI)	Distal intermediate level HRa (95%CI)	Proximal intermediate level HRa (95%CI)	Proximal level HRa (95%CI)
Maternal white skin color	**1.46 (1.23–1.73)**	**1.31 (1.10–1.56)**			
Maternal age < 20 years	0.88 (0.74–1.05)	0.90 (0.74–1.09)			
Maternal schooling ≤8 years	**0.80 (0.70–0.92)**	0.92 (0.78–1.08)			
Primiparous mother	**1.21 (1.05–1.38)**	**1.21 (1.05–1.40)**			
Family income < 2 minimum wages	**0.75 (0.65–0.85)**	**0.81 (0.71–0.94)**			
Attendance to prenatal lecture/course on breastfeeding	**0.84 (0.72–0.99)**	–	0.92 (0.79–1.09)		
Smoking during pregnancy	0.99 (0.63–1.57)	–	1.21 (0.76–1.93)		
Public prenatal care funding	**0.75 (0.65–0.86)**	–	0.90 (0.76–1.06)		
Vaginal delivery	**0.85 (0.74–0.97)**	–	–	1.02 (0.86–1.21)	
Public hospital finding	**0.77 (0.66–0.89)**	–	–	0.90 (0.75–1.09)	
Breastfeeding in the first hour of life	0.96 (0.84–1.10)	–	–	0.99 (0.85–1.15)	
Rooming in at hospital	1.12 (0.87–1.44)	–	–	1.17 (0.90–1.52)	
Received guidance on breastfeeding at hospital	1.12 (0.94–1.33)	–	–	1.11 (0.93–1.33)	
Maternal expectation of breastfeeding duration < 12 months	0.95 (0.80–1.14)	–	–	0.94 (0.79–1.12)	
Birth weight < 2500 g	0.71 (0.51–1.01)	–	–	0.80 (0.56–1.12)	
Infant sex male	1.13 (0.99–1.30)	**–**	**–**	**1.18 (1.03–1.35)**	
Mother working outside the home at the time of breastfeeding interruption	1.07 (0.93–1.24)	**–**	**–**	**–**	**1.52 (1.30–1.77)**
Partner’s support and encouragement to breastfeeding	**0.69 (0.52–0.91)**	–	–	–	0.84 (0.61–1.16)
Cohabitation with child’s father	0.89 (0.74–1.07)	–	–	–	0.81 (0.67–1.00)
Use of a pacifier at the time of breastfeeding interruption	**4.57 (3.96–5.28)**	–	–	–	**3.46 (2.98–4.01)**
Mother-infant bed-sharing at the time of breastfeeding interruption	**0.50 (0.43–0.59)**	**–**	**–**	**–**	**0.61 (0.52–0.73)**
Breastfeeding on demand in the first month	**0.51 (0.37–0.69)**	**–**	**–**	**–**	**0.64 (0.47–0.89)**
Exclusive breastfeeding at 4 months	**0.53 (0.44–0.64)**	**–**	**–**	**–**	**0.58 (0.48–0.70)**

*95%CI* 95% confidence interval, *HRa* adjusted hazard ratio, *HR* hazard ratio

## Discussion

There were a few studies examining the risk factors of breastfeeding interruptions before 2 years of child age [12–16]. Each of these studies showed different risk factors for this outcome. This diversity of results is probably due to the different characteristics of the populations studied, the risk factors explored and the strong influence of socio-economic-cultural factors in the practice of breastfeeding. The present study differs from the previous ones regarding the representativeness of the sample, given that women were selected in all maternity wards in the city, with the exclusion of only a few mother-child pairs. Also, it explored risk factors never explored in those studies, like sleeping environment at the time of breastfeeding interruption. Compared to other two Brazilian studies, the present one was carried out in a region of the country (northeast) with social, economic and cultural characteristics very different from those in the south of the country, where the other Brazilian studies were carried out.

The present results confirm that risk factors differ across populations. Of the 23 variables assessed, five were identified as risk factors, and four as protective factors.

The risk factor with the most robust association with the outcome was use of a pacifier at the time of breastfeeding interruption. The risk of interrupting breastfeeding before 2 years was almost 3.5 times higher for children who used a pacifier. This association was found in two other Brazilian studies that investigated breastfeeding maintenance for 2 years or more [12, 13]. There are at least four arguments to explain this association, all of which may occur simultaneously: the use of a pacifier per se may reduce breastfeeding; use of a pacifier may indicate breastfeeding difficulties; it may be a reflection of the infant’s personality and of mother-infant interaction patterns; and there could be a profile of mothers/families that support both non-use of a pacifier and longer breastfeeding duration [18]. However, it is important to mention that there is no consensus in the literature regarding the recommendation to use or not to use a pacifier. Some investigators contraindicate the use of this artifact due to assumed adverse effects, e.g., reduction of breastfeeding duration [12, 13] and increased risk for otitis media [19], gastroenteritis [20] masticatory dysfunction [21], and distoclusion [22, 23]. Conversely, other authors recommend their use, especially in an attempt to reduce the risk of sudden infant death syndrome [24].

Working outside the home at the time of breastfeeding interruption was the second strongest risk factor for breastfeeding discontinuation before 2 years of age. Of the four studies that were designed to identify determinants of breastfeeding maintenance for 2 years or more and investigated this variable [12–15], two found an association between mother working and breastfeeding interruption before 2 years [12, 15]. One of those studies was performed in Los Angeles and showed that women who returned to work before 3 months postpartum had a lower prevalence of breastfeeding at 2 years when compared to those who returned to work after the 7th month postpartum [15]. The other study, conducted in Brazil, showed that mothers staying at home during the infant’s first 6 months of life contributed to breastfeeding maintenance for 2 years or more [12]. Our study therefore corroborates that maternal employment is a risk factor for breastfeeding maintenance and confirms the need for greater attention to working mothers with regard to providing support and encouragement to breastfeeding, in addition to prioritizing public policies that protect working mothers, e.g. the 6-month maternity leave [15], which would favor infant care and breastfeeding, i.e., activities that require physical contact between mother and infant and that strengthen the bond between them.

Another risk factor found in our study was maternal white skin color. This finding is consistent with other nation-wide studies conducted in Brazil that have shown that women with black and brown (*pardo*) skin color tend to breastfeed for longer [10, 25]. Conversely, two other Brazilian studies failed to show an association between maternal skin color and breastfeeding maintenance for 2 years or more [12, 13]. Still, it is interesting to observe that these latter studies were conducted in the southernmost region of the country, which has different cultural influences when compared to the northeastern region, and a considerably lower number of inhabitants with non-white skin color. Thus, one could argue that these behavioral differences associated with skin color are probably more related to customs, norms, and social traditions, not to mention income level and social network, than to skin color per se.

Primiparity was also identified as a risk factor in the present study. Of the three studies that have explored this variable [13–15], only the Brazilian study involving adolescent mothers reported this association [13]. We speculate that having the experience of having a previous child can help women overcome breastfeeding difficulties and thus contribute to the maintenance of breastfeeding for longer [18].

Finally, male infants were less frequently breastfed for 2 years or more compared to female infants. In the Brazilian study involving adolescents, the frequency of breastfeeding was higher among girls than among boys, but only at 12 months, not at 2 years [13]. The finding of the present study is in line with another previous nationwide study conducted in Brazilian capitals [26] and one study with representative sample of 111 municipalities of the São Paulo State [27], in which female infants showed a higher prevalence of breastfeeding in the first year of life. Some authors discuss the possibility of there being a belief among health-care professionals and/or mothers that boys have greater nutritional needs, requiring complementary feeding from an earlier age [28].

Four protective factors against breastfeeding interruption before 2 years of age were identified: low family income, exclusive breastfeeding at 4 months, on-demand breastfeeding in the first month, and mother sharing the bed with the infant.

None of the four previous studies conducted to investigate determinants of breastfeeding maintenance for 2 years or more [12–15] explored the variable family income; conversely, all of them explored maternal schooling, which can be considered to serve as a proxy for socioeconomic level. Only the study performed in Croatia reported an association, however in opposite direction than the one found here, i.e., women with higher levels of schooling showed a higher chance of maintaining breastfeeding for 2 years or more. Notwithstanding, our finding is compatible with the Brazilian reality. The National Demographics and Health Survey conducted throughout the country showed a higher median of breastfeeding duration among women with up to 4 years of schooling (21 months) when compared with those with more years of formal education (13 months) [10]. Our finding is also in line with the global reality of low- and middle-income countries, i.e., that breastfeeding is more frequent and lasts longer among poorer populations [11].

Longer duration of exclusive breastfeeding had already been shown to contribute to breastfeeding maintenance for 2 years or more in another Brazilian study. Martins et al. reported that, for every additional day without the introduction of water and/or teas, and without the introduction of any other milk to the infant’s diet, the probability of the infant being breastfed for 2 years or more increased by 0.5 and 0.1%, respectively [12]. The explanation behind this association could be related to different factors: the decrease observed in milk supply when the infant consumes less breast milk as a result of being given other foods; the possibility of nipple confusion, as many of the foods offered along the first 6 months (i.e., when exclusive breastfeeding is recommended) are offered using a bottle [29]; or even, a maternal profile that supports both compliance with the recommendation of duration of exclusive breastfeeding and any breastfeeding.

The other two factors found to protect breastfeeding maintenance for 2 years or more, namely, breastfeeding on demand in the first month and mother sharing bed with infant, had not been described to date.

On-demand breastfeeding is related with enhanced milk supply [2, 9], which in turn facilitates breastfeeding maintenance. Conversely, the association between mother-infant bed-sharing and longer breastfeeding duration can be explained by the increased physical contact between mother and infant and the easy access to breastfeeding at night [30]. However, there is no consensus recommendation regarding bed-sharing due to the possibility of an increased risk of sudden infant death syndrome when the infant shares the bed with their parents, especially in the first 3 months of life [24].

Rollins et al. [31] elaborated a three-level conceptual model for the determinants of breastfeeding: structural (sociocultural and market context), settings (health systems and services, family and community and work place and employment) and individual (mother and infant attributes and mother-infant relationship). It is interesting to note that most of the determinants of the interruption of breastfeeding before the age of 2 in the population studied are related to the attributes of the mother-infant pairs and only one is related to setting (maternal work) and none related to health services. The explanations for these findings requires additional studies.

Among the five studies that assessed risk factors for weaning before the age of 2, three also investigated factors associated with interruption of breastfeeding in different time points - 6, 12, and 24 months. The results showed that the factors associated with breastfeeding duration may vary depending not only on the population studied, but also on the time frame assessed. Only one factor in the Croatian study (antenatal course attendance) and in one of the Brazilian studies (pacifier use) were associated with breastfeeding maintenance at the three time points assessed. This fact has practical implications for the promotion, protection and support of breastfeeding.

The main limitation of the present study was the great number of cases lost during follow-up. However, this is a common problem in cohort studies, especially when the follow-up period is long, as a result of population mobility. In addition, when comparing the characteristics of the participants with those who did not complete the follow-up, it was observed that there was no statistically significant difference for most of the variables, suggesting that the losses did not cause additional bias to the measures of association of the final model [32].

Also, a few variables were measured at the time of breastfeeding interruption, preventing the establishment of the causality between these variables and the outcome. In addition, the study did not explore structural determinants as social trends, advertising, media, interventions at the structural level, legislation, policy, and media and social mobilization. Conversely, the strengths of the study include its design (livebirth cohort with mother-infant pairs selected at all maternities of the city with 2-year mother-infant follow-up) and the hierarchical analysis of data, which enabled to observe that among the socioeconomic and demographic factors that influence breastfeeding duration, those closest to the outcome were the most relevant ones. Another positive characteristic of the study was minimization of memory bias, as data collection on the infant’s feeding habits was conducted monthly in the first 6 months and then at 9, 12, 18, and 24 months of age, always focusing on the previous 24 h.

## Conclusions

Distinct socioeconomic/demographic variables and post-discharge behavioral characteristics were identified as either risk factors for and protective factors against breastfeeding interruption before 2 years of age. Some of these factors had already been reported in the literature, and others are new, pointing to differences across populations. Moreover, several of the factors associated with the outcome in this study are modifiable, which reinforces the importance of adopting public intervention policies and implementing measures against the factors that hinder breastfeeding and supportive of the factors that contribute to this practice, so that the international recommendation to breastfeed for 2 years or more can be successfully implemented. The identification of the factors associated with interruption of breastfeeding before 2 years for the majority of the live births in Feira de Santana adds new and relevant information that can be used for the prevention of breastfeeding abandonment before 2 years and for the proposition of actions to promote, protect, and support breastfeeding maintenance as recommended by the WHO in that population.

## Data Availability

The instruments used, the data collected and the results of the post-analysis were stored in the NUPES / UEFS for 5 years, according to the guidance of Resolution 466/12. The data are being made available upon request and authorization from the responsible researchers. The eligibility rules for using the data and authorship, and any intention of using the professional writers, will occur through the respective participation of each team member in the study and authorization from the responsible researcher.

## Abbreviations

95%CI: 95% confidence interval
HR: Crude hazard ratio
HRa: Adjusted hazard ratio
SPSS: Statistical Package for the Social Sciences
WHO: World Health Organization
